# Novel Strategies for Malaria Vaccine Design

**DOI:** 10.3389/fimmu.2018.02769

**Published:** 2018-11-29

**Authors:** Augustina Frimpong, Kwadwo Asamoah Kusi, Michael Fokuo Ofori, Wilfred Ndifon

**Affiliations:** ^1^Department of Biochemistry, Cell and Molecular Biology, West African Centre for Cell Biology of Infectious Pathogens, College of Basic and Applied Sciences, University of Ghana, Accra, Ghana; ^2^Immunology Department, College of Health Sciences, Noguchi Memorial Institute for Medical Research, University of Ghana, Accra, Ghana; ^3^African Institute for Mathematical Sciences, Cape Coast, Ghana; ^4^African Institute for Mathematical Sciences, University of Stellenbosch, Cape Town, South Africa

**Keywords:** *Plasmodium falciparum*, malaria, vaccine, immunoinformatics, structure-based, lymphocyte repertoire sequencing

## Abstract

The quest for a licensed effective vaccine against malaria remains a global priority. Even though classical vaccine design strategies have been successful for some viral and bacterial pathogens, little success has been achieved for *Plasmodium falciparum*, which causes the deadliest form of malaria due to its diversity and ability to evade host immune responses. Nevertheless, recent advances in vaccinology through high throughput discovery of immune correlates of protection, lymphocyte repertoire sequencing and structural design of immunogens, provide a comprehensive approach to identifying and designing a highly efficacious vaccine for malaria. In this review, we discuss novel vaccine approaches that can be employed in malaria vaccine design.

## The global malaria situation

Malaria caused by *Plasmodium* parasites remains a major infectious disease of public health importance. The disease is caused by five protozoan species, namely *Plasmodium falciparum, P. vivax, P. malariae, P. ovale*, and *P. knowlesi*. The deadliest of these is *P. falciparum* which is predominant in sub-Saharan Africa (SSA). In 2016, approximately $2.7 billion was invested globally in control and elimination programs (1). Meanwhile, it was estimated in 2016 that nearly half of the world's population was at risk of infection, with 91% of the estimated deaths being in Africa and 70% of the mortality occurring in children under 5 years (1). Notwithstanding, preventive control and intervention measures have helped decrease the burden between 2000 and 2015. For instance, the incidence of new malaria cases was down by 37% world wide and 42% for the WHO African region. In addition, the incidence of mortality over the same period decreased by about 60% globally and 66% for the African region (2). Yet, malaria imposes huge economic losses for people in the African Region and there is a need to upscale the available interventions and introduce new ones such as a licensed cost-effective vaccine (3).

## Challenges to the eradication of malaria

Malaria eradication faces many challenges including insecticide resistance, emerging anti-malarial drug resistance and the presence of asymptomatic and submicroscopic infections.

Indoor residual spraying (IRS) and long-lasting insecticidal nets (LLINs), have been among the most effective tools for malaria control and elimination (4). So far, pyrethroids are the only recommended class of insecticides for LLINs. However, more than 30 countries have reported resistance to pyrethroids, which has the potential to spread to new areas (5–9).

The rapid development of pyrethroid resistance suggests that alternative classes of insecticides need to be identified. As a result, WHO has cautioned against the use of pyrethroids (8), raising the need for alternative measures of control. The development of resistance to malaria drugs by *P. falciparum* remains a major threat to malaria elimination. The WHO-recommended first line treatment for uncomplicated malaria caused by *Plasmodium falciparum* is the artemisinin-based combination therapies (ACTs). Historically, *P. falciparum* has been able to develop resistance to almost all previous first-line antimalarial drugs (10, 11). The development of resistance to these drugs almost always begins from South-East Asia, where mutant parasites resistant to antimalarial drugs are more likely to survive due to lower levels of acquired immunity, poor adherence to administered drugs and higher parasite burdens (11–14). *P. falciparum* resistance to artemisinin-based drugs seems to have emerged sporadically (15), with mutations for resistance found within the kelch 13 propeller gene (15, 16). An inevitable fact is that artemisinin resistance may be imminent and other intervention avenues such as the development of highly effective vaccines need to be rapidly explored.

Also, the presence of asymptomatic and submicroscopic infections poses a major threat to malaria eradication and control. Continuous exposure to infectious mosquito bites leads to the development of anti-disease and anti-parasite immunity. The level of this immunity is determined by the transmission intensity and epidemiology of the disease (17, 18). It has been shown that the microscopic prevalence of malaria is almost half of that detected by nucleic acid amplification techniques and lower in low transmission areas (19, 20). The prevalence of submicroscopic infections has been found to be high in low transmission areas and common in children, probably as a result of a less robust immune response, leading to insufficient time for the development of protective immunity. In addition, asymptomatic infections may persist for several months and serve as a major threat to malaria eradication (21) as they sustain disease transmission (22–25).

## Current approaches to developing a malaria vaccine

### Malaria vaccines

The acquisition of partial immunity and the successful treatment of clinical symptoms of malaria in children with purified immunoglobulins from semi-immune adults (26) are positive indications of the feasibility of a vaccine against malaria. This is also supported by the induction of sterile immunity in both animal models and controlled human malaria infection (CHMI) through immunization with either live or attenuated sporozoites and merozoite-infected red cells (27–29). Attenuated sporozoites, even though they still maintain their natural hepatocyte invasion ability, do not fully mature in the liver and hence do not form merozoites that are responsible for the clinical symptoms of malaria (30).

### Vaccine targets

There are three stages to target for a potential malaria vaccine candidate. The first target of vaccine development is the pre-erythrocytic stage. This is the period where sporozoites travel through blood and infect hepatocytes to undergo schizogony, the vigorous multiplication stage that precedes the invasion of red blood cells (RBCs). The main purpose of developing a vaccine against this stage is to inhibit hepatocyte infections and hepatic parasite development, thus limiting RBC invasion (27, 30). The mechanisms of protection for this stage may involve antibody responses that prevent sporozoites from invading hepatocytes or cytotoxic T cells that destroy infected liver cells. So far, the licensed RTS,S, subunit vaccine remains the most advanced malaria vaccine to be developed. Other candidate vaccines include the whole-parasite vaccine candidates such as *Pf* sporozoite (PfSPZ), PfSPZ vaccination with chemoprophylaxis (PfSPZ-CVac) and the genetically attenuated parasite (PfSPZ-GAP).

The second target for malaria vaccine candidate design is the blood-stage of the parasite. The motivation for developing such vaccine candidates comes from evidence that people with repeated malaria infections in endemic areas develop some level of protective immunity, a state in which there is immune-controlled RBC invasion, resulting in fewer disease symptoms or asymptomatic infections (26, 31). Accordingly, vaccine candidates have been designed to elicit immune responses that will block/limit merozoite invasion of RBCs and stop the rapid replication of merozoites by targeting parasite surface proteins such merozoite surface proteins, apical membrane antigen 1 (AMA1), and the reticulocyte homolog (Rh) proteins (32–35). Other blood-stage vaccines target parasite antigens embedded in infected RBC membranes, such as *P. falciparum* Erythrocyte Membrane Protein-1 (PfEMP1) (36).

Despite being highly immunogenic and showing good promise as vaccine candidates, most of these antigens are also highly polymorphic and hence elicit antigen and parasite strain-specific responses (32, 33). Conversely, antigens such as the Rh proteins that show a high level of conservation (34, 35) tend to be less immunogenic (37).

The third malaria vaccine candidate target is the sexual parasite forms or gametocytes. Malaria transmission-blocking vaccines (TBVs) are designed to interrupt parasite transmission between humans and the mosquito vector through host immunological response to parasite targeted proteins such as Pfs230, Pfs45, Pfs48 (pre-fertilization antigens) and Pfs25, Pfs28 (post-fertilization antigens). Successful malaria transmission depends on the availability of infectious gametocytes in human peripheral blood that can be taken up by mosquitoes during a blood meal. Studies have reported that the degree of infectivity of gametocytes to mosquitoes is based on the gametocyte density, drug stress, clonality of infection and immune defenses of the mosquito (38–42). However, according to Churcher et al. (38), even at very low densities, gametocytes remain infectious to mosquitoes. Also, it has been reported that in the human host, transmission can be stable at very low densities and is not directly proportional to the gametocyte density in peripheral blood (43, 44). Basically, a TBV exploits the fact that there is a functional immunological activity against the sexual stage parasite proteins which is able to reduce the infectivity of the parasite, thereby decreasing malaria transmission (45, 46). Vaccine candidates that seek to interrupt malaria transmission (VIMT) are of two main types: (1) sexual, sporogenic or mosquito stage VIMT (SSM-VIMT) candidates which are expected to interrupt human-to-mosquito transmission; and (2) the pre-erythrocytic VIMT (PE-VIMT) candidates, which are expected to interrupt mosquito-to-human transmission (47). Among the TBV candidates, only Pfs25 and Pfs230 have undergone clinical trials in human (48–51). Unfortunately, a major challenge with these candidate vaccines is the inability to elicit higher antibody titers. In regards, there are considerations to conjugate these candidate vaccines (50, 52).

### Current status of malaria vaccine development

After decades of extensive research, the pre-erythrocytic stage vaccine, RTS,S has been licensed and is expected to undergo further testing in malaria endemic areas before possible approval for immunization. Currently, together with RTS,S, only 20 candidate vaccines are undergoing clinical trials (Table 1). For RTS,S, a recent evaluation on the safety and immunogenicity of the vaccine co-administered with the recommended expanded programme on immunization showed the vaccine to be safe and immunogenic with no related adverse events (58). The RTS,S/AS01 consists of a recombinant protein of the *P. falciparum* circumsporozoite protein (CSP) conjugated to a hepatitis B virus surface antigen. During clinical trials, the efficacy of the vaccine after 4 doses was observed to be 43.9% in children aged 5–17 months and 27.8% in children 6–12 weeks old (59). However, vaccine efficacy wanes with time and fails to meet the target set by the Malaria Vaccine Technology Roadmap (60). Consequently, other vaccination regimens such as the number of doses, time of immunization, and alternative approaches for vaccination are being evaluated (61).

**Table 1:** Current malaria vaccines in clinical trials.

**Vaccine candidate**	**Clinical trial registration number**	**Clinical trial stage**
**PRE-ERYTHROCYTIC**		
RTS,S/AS01	NCT01345240	Phase 3
R21/AS01B	NCT02600975	Phase 1
R21/ME-TRAP	NCT02905019 (53)	Phase 2
ChAd63/MVA ME-TRAP	NCT01635647 (54–56)	Phase 2
R21/Matrix-M1	NCT02572388/NCT02925403	Phase 1/2
PfSPZ Vaccine	NCT03510481	Phase 1
PfSPZ-CVac (PfSPZ Challenge + chloroquine or + chloroquine/pyrimethamine	NCT03083847	Phase 1
GAP 3KO (52-/36-/sap1-)	NCT02313376	Phase 1
**BLOOD-STAGE**		
pfAMA1-DiCo	NCT02014727 (57)	Phase 1
P27A	NCT01949909	Phase 2
PAMVAC	NCT02647489	Phase 1
PRIMVAC	NCT02658253	Phase 1
**SEXUAL-STAGE**		
ChAd63 Pfs25-IMX313/MVA Pfs25-IMX313	NCT02532049	Phase 1
Pfs25-EPA/Alhydrogel	NCT01867463, 51	Phase 1
Pfs230D1M-EPA/Alhydrogel and/or Pfs25-EPA/Alhydrogel	NCT02334462	Phase 1
Pfs25M-EPA/AS01 and/or Pfs230D1M-EPA/ASOI	NCT02942277	Phase 1
Pfs25 VLP-FhCMB	NCT02013687	Phase 1
Pfs25-Pfs25	NCT00977899	Phase 1
Pfs25 & Pvs/Monatide ISA 51	NCT00295581	Phase 1

*Adapted from WHO. 9/28/2018. Malaria Vaccine Rainbow Tables. http://www.who.int/vaccine_research/links/Rainbow/en/index.html*.

Also, the R21, a virus-like particle vaccine which is a biosimilar of RTS,S consists of the CSP conjugated to a single hepatitis B surface antigen. The RTS,S-like vaccine has been shown to provide sterile protection in mice at very low doses. In addition, it was observed that most of the immune responses elicited against the candidate vaccine targeted the CSP in contrast to the hepatitis B surface antigen which is often targeted in the RTS,S vaccinated individuals (53). Importantly, this candidate vaccine is designed such that more epitopes of the CSP may be exposed to host immune system to enhance the efficacy of R21.

Furthermore, the whole sporozoite vaccine has been reported to provide significant protection against falciparum malaria. The whole organism candidate vaccine design approaches include the radiation-attenuated sporozoites (PfSPZ), whole PfSPZ with chemoprophylaxis (PfCVac) and the genetically modified sporozoites (PfGAP). Even though PfCVac showed complete protection to homologous *P. falciparum strain*, moderate protection has been observed with heterologous strains in non-exposed vaccines (62). Clinical trials with PfSPZ in endemic areas have been shown to be safe and well tolerated, however, inducing low level of immune responses compared to naïve individuals (63, 64). These may suggest that the breadth of immune responses to PfSPZ vaccines need to be increased by considering other vaccination regimens.

## Promising approaches to malaria vaccine development

Recent technological advances have greatly improved the prospects for designing an effective malaria vaccine through advances in high-throughput biology and computation. These alternative approaches may be focused on the parasite- or host immune system.

### The parasite-focused approach

The technologies involved in this approach center on the identification of immunogenic antigens from the pathogen by interrogating the parasite's genome, transcriptome or proteome. It may modify the structure of the antigenic component(s) identified with the aim of targeting various strains of the pathogen. The parasite-focused approach further tests the immunogenicity and safety of the candidate antigens to design novel and improved vaccines. This approach may involve the application of reverse vaccinology, structural vaccinology, and immunoinformatics.

### Reverse vaccinology

**Figure 1:**
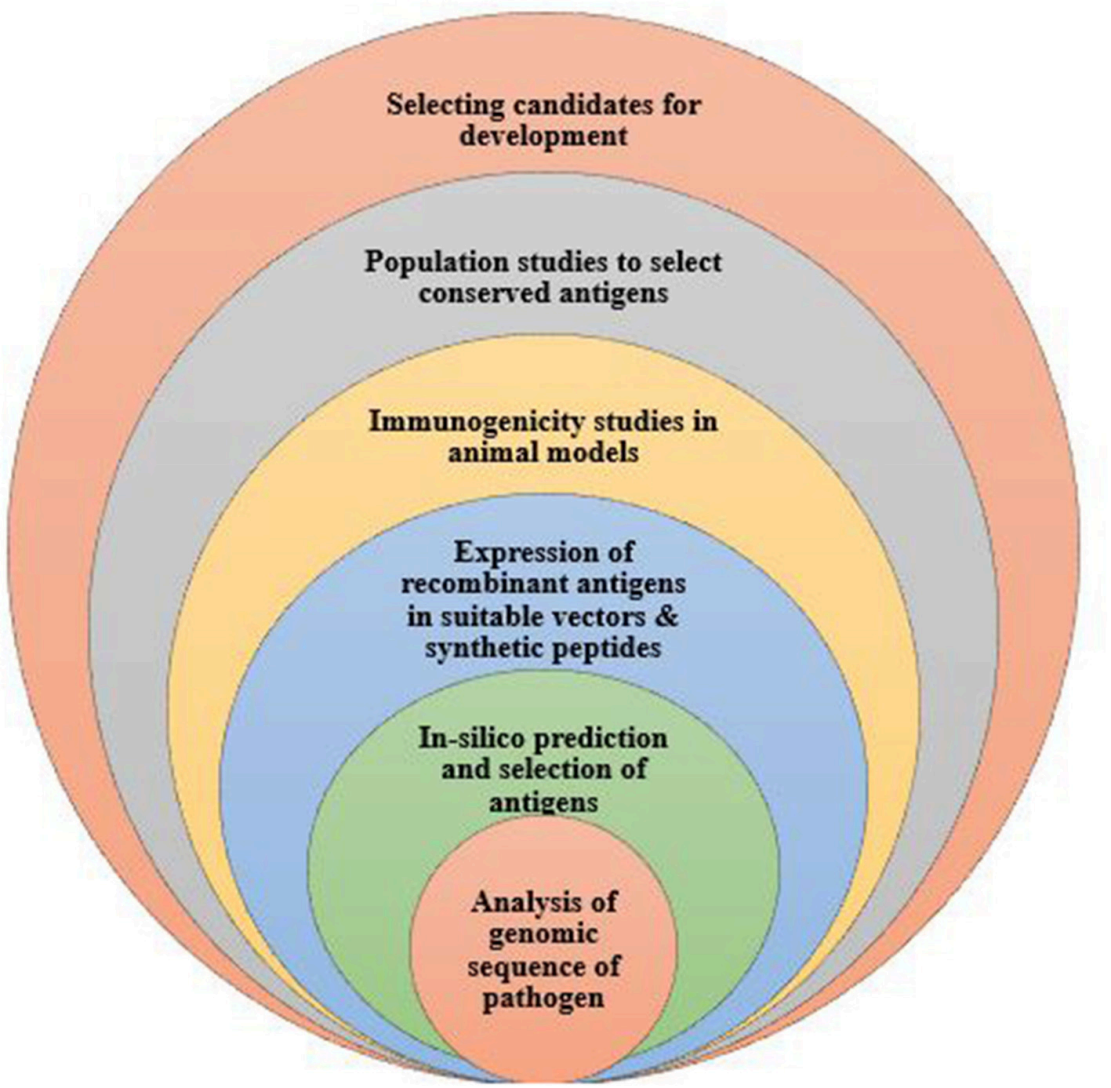
The process of developing a vaccine using reverse vaccinology. Reverse vaccinology starts with obtaining the genomic sequence of the pathogen and using bioinformatics tools to identify all open reading frames to predict protein antigens. The predicted antigens are subsequently expressed in suitable vectors to produce the recombinant proteins which are tested to evaluate the immunogenicity in animal models. Expressed antigens that yield high immunogenicity are selected as vaccine candidates, further tested in population studies to determine and identify conserved antigens for further vaccine development.

Reverse vaccinology, developed by Rappouli et al. is a technology first used in *Meningococcus* serogroup B bacteria to identify novel vaccine antigens (Figure 1). Here, the pathogen's genome is sequenced and analyzed to have access to the entire repertoire of proteins and enable comparison of conserved sequences shared among pathogens of the same species (65). Genomic data is analyzed using bioinformatics tools, taking into consideration all open reading frames. Also, with the use of computational tools, genomic sequences that are homologous to those of humans are eliminated from the vaccine candidates identified. The remaining genes are isolated and inserted into a suitable vector to obtain proteins for testing in animal models. Responses to the vaccine antigens are analyzed in immunized mice to validate their immunogenicity and efficacy levels. Importantly, molecular epidemiology studies are undertaken using various strains of the pathogen to ascertain whether the selected antigens are conserved or highly variable in a given population (66). This approach has been used to develop vaccines against serogroup B *Neisseria meningitidis* (67); and identify vaccine candidates for, *S. agalactia* and *S. pyogenes* (68, 69). This vaccine design approach has greatly enhanced the discovery and characterization of several pathogen antigens.

Reverse vaccinology has been applied in malaria to identify parasite proteins either secreted or involved in signaling for consideration as possible vaccine candidates. The genomic sequence of *P. falciparum* has been available since 2002 (70). In addition, the sequence of other diverse *Plasmodium spp* including primate (71, 72) and rodent (73) parasites have been published. Comparative analysis of these sequences has shown similar homologs between species with possibly similar functions. For instance, the conserved Pf48/45 and PfHAP2 genes, both of which are transmission blocking vaccine candidates, functions were determined based on the role of their homologs in other *Plasmodium* spp. The functional analysis of the P48/45 and PHAP2 genes in *P. berghei* established their significant role in reducing the fertility of male gametes during fertilization, promoting these genes as vaccine candidates (74, 75), which are currently in the preclinical stage (76–78).

Despite the success of reverse vaccinology, it cannot be used to identify non-peptide antigens but can identify operons that code for synthesis of such molecules (79). For pathogens with complex genomes such as malaria parasites, no successful vaccine has as yet been developed via this approach. Further progress requires, among other things, improved predictive algorithms to identify the T and B cell epitopes as well as accurate quantitative assessments before inclusion in vaccines.

### Structural vaccinology

An improved understanding of the native structures of biological macromolecules such as proteins and how changes in their structure affect their functions can assist the identification of suitable epitopes (80, 81). Such epitopes can be designed into accessible forms for easy uptake by immune cells. These structural considerations make it possible to improve vaccine immunogenicity and safety and mitigate the effects of sequence variability within different strains of a pathogen (82). For instance, the bacterium *Meningococcus* is able to evade the host's immune system with the aid of a factor H binding protein (Hbp), which inactivates the host complement pathway by blocking factor H. Structural considerations allowed immunodominant epitopes of Hbp from various meningococcal strains to be identified and grafted into a single variant molecule to form a single antigen. This antigen was used in the MenB vaccine, responses against which are able to neutralize all the targeted strains (83).

Also, in an earlier study, short conserved α-helical coiled coil structural domains were identified from the asexual blood stage of the *P. falciparum* by examining the *Plasmodium* genome (84). Upon further screening, an unstructured peptide (P27A) that unfolds in native confirmation was selected. The peptide was the target of human antibodies which were able to restrict parasite replication (85). The vaccine candidate P27A has been considered immunogenic and safe with mild adverse events after Phase1 clinical trials (86).

For pathogens like *P. falciparum*, structural vaccinology may also help overcome antigenic variation. For instance, the application of structural vaccinology enabled the characterization of the less polymorphic DBL4ε domain of VAR2CSA to identify novel properties in the motif that affects the functional features of the antigen (87); identification and confirmation of the three-dimensional structure of the invasion ligand Cysteine-Rich Protective Antigen (CyRPA) (88). For example, the CyRPA was identified as a protective epitope providing an additive effect with the Reticulocyte binding-like Homologous protein 5 (*Pf* RH5) such that antibodies against *Pf* RH5 and CyRPA can inhibit parasite replication in host RBCs (88). Hopefully malaria vaccines incorporating these epitopes may elicit strong protective immune responses. Combining these protective antigens to create hybrid protein vaccines with enhanced efficacy may be a viable option for malaria.

A key challenge with this approach is the identification of suitable B and T cell epitopes for incorporation into vaccine candidates.

### Immunoinformatics based approach to vaccine design

Immunoinformatics integrates both computational approaches and experimental immunology to develop machine learning algorithms that attempt to predict the immunogenicity of antigens. These approaches can be either pattern- or theory-based and may operate at either the amino acid sequence or the protein structure level. The pattern-based approaches conceive the prediction problem as one of finding sequence/structural patterns associated with immunogenicity. In contrast, the theory-based approaches attempt to model the basis for immunogenicity, for example, by using physical principles. Examples of algorithmic tools employed by pattern-based approaches include quantitative structure-activity relationship analysis, support vector machines, and artificial neural networks (89, 90). Theory-based approaches often employ Markovian and/or Bayesian models as well as models based on statistical mechanics (91).

Immunoinformatic approaches have already been applied to *P. falciparum* to predict possible cytotoxic T cell epitopes coupled with HLA A/B molecules for malaria peptide vaccine design (92). For example, the PfEMP1 gene, a member of the var gene family has been associated with parasite evasion from host immune mechanisms due to its multiple variation and ability to bind to different host receptors (36). In a recent study, both *in-silico* and experimental approaches were used to identify antigenic epitopes from CIDR-1 and DBL-3γ conserved domains of PfEMP1. These epitopes were predicted to have good binding affinity to HLA molecules as well as the capability to induce IFN-γ, IL-4 secretion and T cell proliferation in exposed individuals (93).

Classically, HLA class I molecules optimally require peptides that are 8-10 amino acids long for presentation to CD8 T cells while HLA Class II molecules optimally require 12-25 amino acids long peptides for presentation to CD4 T cells. Of note high predictive accuracies have been achieved for bioinformatics methods for predicting peptide binding to HLA I molecules; whereas those for predicting binding to HLA II molecules require further improvement. An even greater challenge is prediction of peptide binding to B cell receptors for effective antibody responses. On-going work by us and other groups is aimed at addressing some of these challenges (91, 94). However, not all HLA binders are good epitopes for T cells and this poses a major challenge for approaches that predict HLA binders without considering the global picture of HLA-peptide-TCR interactions. Nonetheless, these computational approaches, which are quite cost-effective and are important down-selection tools in instances where there are too many peptides to evaluate experimentally, have the potential to aid in the development of effective vaccines against malaria.

### Immune-focused approach

**Figure 2:**
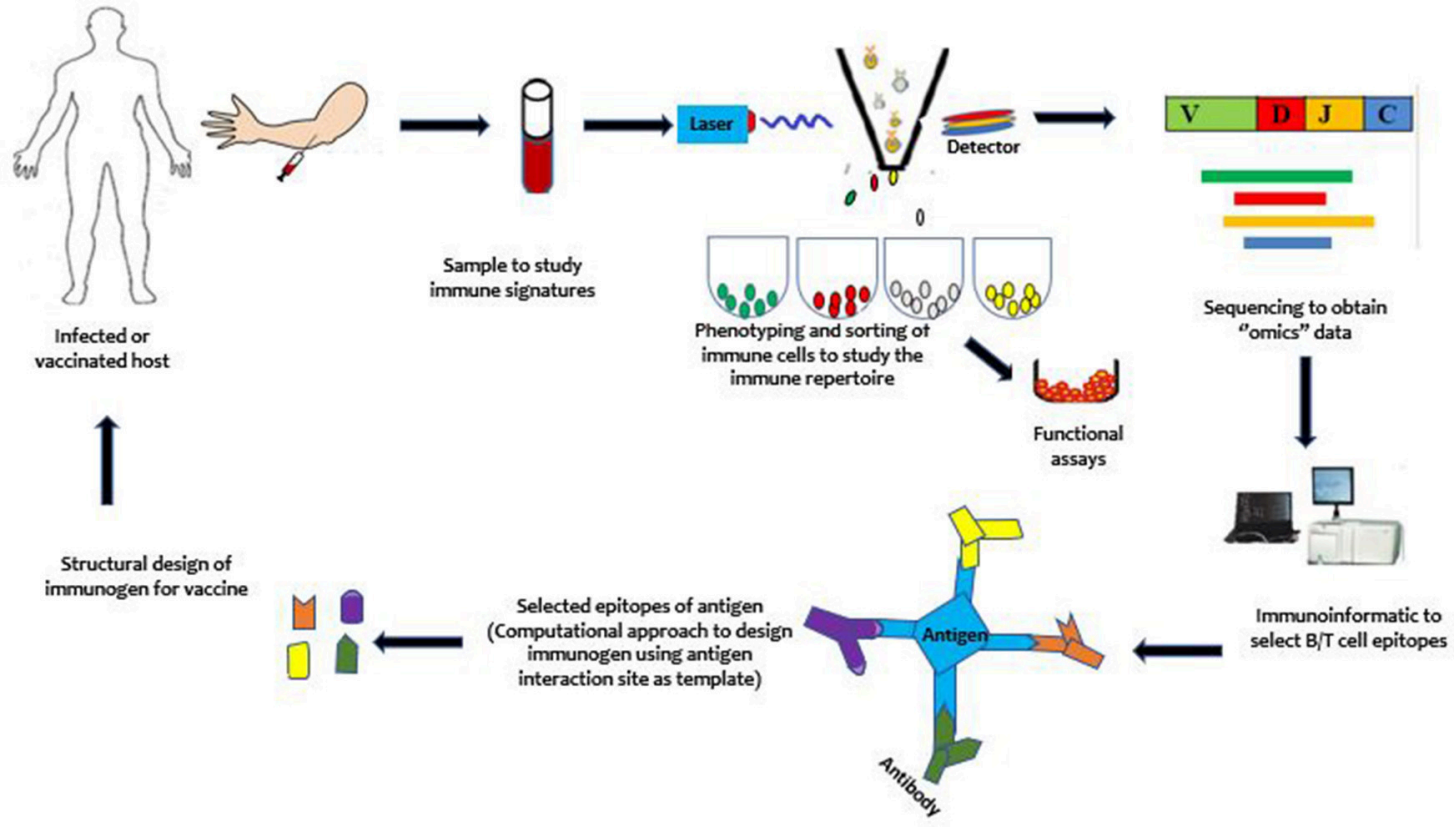
An illustration of the immune-focused approach to vaccine development. Briefly, from top left, samples are obtained from an infected but protected or a vaccinated host. Immune cells are phenotyped and sorted using single cell sorting technologies such as the flow cytometry. The sorted cells may be sequenced directly or proliferated for functional studies before sequencing to study the immune repertoires (B/T cell receptor repertoire). With the use of computational approaches, the immunogen is designed and taken through further stages of development to yield a vaccine. After vaccination, mechanisms underlying vaccine efficacy and safety can be studied to further enhance the developed vaccine to achieve maximum protection against the targeted pathogen.

Due to the sophisticated immune-evasion mechanisms of *P. falciparum* that allow it to coexist with the host, vaccinologists require new paradigms in vaccine development. One such new paradigm that has been developed to target these pathogens is the immune-focused approach (Figure 2). In contrast to the parasite-focused approach, which centers on the pathogen of interest, this new approach seeks to harness the host immune system to more rapidly design effective vaccines. It focuses on studying the host immune system to discover protective immune signatures. It is expected that these protective signatures can be induced *de novo* in susceptible hosts to protect them against infection and/or disease. Compared to the pathogen-focused approach, the immune-focused approach has, in principle, a greater potential for success against pathogens like malaria parasites, which have highly variable genomes. In particular, it may be able to identify and design immune cells with broadly neutralizing antibodies (95) and enhanced cellular immune responses, which has proved difficult to accomplish by using conventional approaches. To provide context for the discussion of opportunities for vaccine development, we begin with a brief overview of human immunity to malaria.

#### Immunity to malaria

In contrast to many pathogens against which highly potent, long-lived immunity is achieved, human immunity to malarial parasites is less potent and relatively short-lived (17). In malaria, acquired immunity to infection is rare; rather, what develops naturally, generally over a long period, is acquired immunity to disease (96). Such clinical immunity generally targets the disease-causing asexual blood stage of malarial parasites. It tends to be acquired faster in moderate-to-high transmission settings compared to low transmission areas, and with a higher number of clinical episodes (97–99). As with other infectious diseases, the development of clinical immunity to malaria is dependent on the adaptive arm of the immune system, and the principal mediators consist of specific subsets of B and T cells. Some progress has been made to elucidate the underlying mechanisms, although the key immune determinants remain unclear.

In addition, the ability to predict the beginning and end of transmission seasons have made it possible to study host responses to infection and some immune dynamics that occur before, during and after infection as well as, drug interventions (100) and how they may affect the immunity acquired (101, 102).

In natural infections, acquisition of immunity to sporozoite stage infections is limited, probably due to the low number of sporozoites that are inoculated as well as the limited time that sporozoites have extracellular, prior to hepatocyte invasion. In addition, it has been reported that *Plasmodium* sporozoites are able to modulate the cytokine environment by downregulating Th1 responses and antigen presentation to T cells (103). Recently, it was reported that continuous exposure to *P. falciparum* leads to the induction and expression of immunoregulatory cytokines such as IL-10 and affects the function of dendritic cells (104). These, coupled with frequent infection and immune activation, may profoundly impact on the tolerogenic environment leading to the escape of sporozoites from immune cells. Nevertheless, functional properties of antibodies to sporozoite-stage infections have been associated with natural protection from clinical disease. It has been reported that these antibodies kill sporozoites through complement fixation and inhibit hepatocyte invasion. However, the response to sporozoite antigens was age-dependent and acquired slowly compared to blood-stage antigens (105).

The blood-stage parasite is associated with the clinical symptoms of the disease as it causes an upregulation of pro-inflammatory cytokines, regulatory T cells and parasite sequestration in small blood vessels in host organs. Antibodies have been reported to play functional roles in preventing parasite invasion of red blood cells (106, 107). Antibodies to parasite antigens are associated with clinical immunity in endemic areas (108–110). The mechanisms of antibody activity may include blocking invasion of erythrocytes (111); opsonizing parasites to facilitate their clearance (110, 112) enhancing the killing of infected cells by monocytes (113); complement-mediated lysis of infected cells (114); and inhibiting adherence of infected erythrocytes to vascular endothelium (115). However, the generation of atypical memory B cells which have reduced effector functions has been observed under chronic conditions (116, 117).

T cells have also been shown to play protective roles during blood-stage infection. For instance, protection from the disease has been associated with FOXP3^−^ Th1 cells which are self-regulatory and produce IFNγ, TNF and IL-10 (101, 118, 119). These cells are believed to prevent the production of pyrogenic factors that may lead to the manifestation of clinical disease. However, these immune responses are not long-lasting and easily decay after infection has waned. T cell responses are hampered by the upregulation of negative immune regulatory receptors which may blunt or cause anergic responses (116, 120, 121). Our recent study found higher levels of T regulatory cells to be associated with higher blood levels of *P. falciparum* in children (118), suggesting less effective control of the parasite. Indeed, trying to understand these various aspects of the immune responses is a quite complex task (122).

Compared to natural infections, inducing sterile immunity in naïve individuals has been achieved through whole sporozoite immunization (29, 123) although similar outcomes have not been seen in individuals from malaria endemic areas (63, 64). Vaccination of volunteers with radiation-attenuated sporozoites has shown that both T cells and antibody responses play a significant role in protecting vaccinated cohorts against clinical challenge. It was observed that T cells from the periphery of these individuals, when stimulated with *P. falciparum* sporozoites *in vitro*, produced effector cytokines in a dose-dependent manner whereas antibody levels increased and prevented hepatocyte invasion (124–127).

The challenges of inducing immunity to malaria by natural or artificial means are compounded by the sophisticated immune-evasion strategies of the parasite. The parasite has a large genome consisting of about 5,300–5,500 possible antigenic targets (70). This extensive gene repertoire coupled with the parasite's high mutation rate allows for extensive variation of antigens that can be potential vaccine targets. Moreover, the epitopes targeted by the immune system exhibit a hierarchy of immunogenicity, with immunodominant epitopes that induce large amounts of antibodies, not all of which are neutralizing and may mask sub-dominant epitopes bound by neutralizing antibodies (73). In addition, the parasite switches off antigenic phenotypes, associated with the variant antigens resulting in functional diversity. Consequently, infections are mostly characterized by successive parasitemia waves caused by different parasite variants, making the development of long-lived immunity to the parasite very challenging (36, 128). Furthermore, key antigens such as CSP contain tandem repeats that have been implicated in immune evasion by suppressing antibody responses against adjacent antigens (129).

### High throughput identification of immune correlates of protection

The age and genetics of a person may modulate the immune responses elicited during infections and vaccinations (130, 131). Nonetheless, these responses that modulate infection may help to systematically define factors associated with protection from disease. Conventional approaches to understanding immune correlates of protection against *P. falciparum* includes, but is not limited to, ELISA, Elispot and Western blots. However, recent advances in high throughput assays have allowed in-depth analysis of immune correlates of protection to multiple *falciparum* antigens. Individuals in malaria-endemic areas generate antibodies to different *P. falciparum* proteins which may be protective or serve as a serological marker for exposure.

High throughput assays that probe the genomic, proteomic and transcriptomic data of immune responses are useful means of determining correlates of protection in exposed and vaccine trial cohorts. Independent studies using library expression and protein microarray has characterized host immune reactivity to different *P. falciparum* antigens. Using these approaches, Doolan et al. (132) were able to identify stage-specific *P. falciparum* antigens associated with protection in naturally exposed individuals, vaccine protected and non-protected individuals using a protein microarray chip with 250 proteins. They observed distinctive antibody profiles in the various groups to these antigens. Also, in an independent study, involving a large cohort of children naturally exposed to malaria (≤10 years old) in Kenya, it was observed that responses to fewer proteins from the 39 *P. falciparum* antigens analyzed were significantly associated with protection, and these included AMA1 and MSP2. Also, antibodies to the top 10 proteins provided an additive effect whereas most antibody responses to the other antigens were markers of malaria exposure (133).

A similar study conducted in Mali probed sera from malaria-exposed children and adults against 1204 proteins. Among these proteins, 91 were associated with sexual stage-specific immunity with specific-IgG responses culminating during the transmission season. It was further observed that immunity to these sexual stage vaccine candidates (Pfs48/45 and Pfs230 but not Pfs25) can be boosted in natural infections (134). These studies showed evidence that the breadth and magnitude of the antibody response is a better correlate of immune protection. Furthermore, in analyzing PBMCs for non-humoral immune responses associated with protection using DNA microarrays, qRT-PCR and flow cytometry, it was observed that repeated exposure to malaria in children was associated with the upregulation of genes involved in immune regulation (such as IL-10 secretion from CD4+Foxp3-), phagocytosis and activation of adaptive immune system. In contrast, gene expression levels of chemokines and cytokines associated with fever and inflammation (such as IL-1β, TNF, CXCL2 and IL-8) were downregulated (101).

Interestingly, the application of next-generation sequencing techniques such as lymphocyte immune repertoire sequencing, including T cell receptor (TCR), membrane-bound B cell receptor (BCR) or secreted BCR can allow an in-depth analysis of host factors associated with pathogen recognition, identification and protection from disease. The TCR structure is heterodimeric with two protein subunits; an alpha and beta chain or gamma and delta chain with both a constant and variable region. Similarly, the BCR consists of two heavy and light chains which are joined together by disulphide bonds to form a Y shaped immunoglobulin together with a variable and a constant region. The lymphocyte receptors (TCR/BCR) have similar structures including a variable, diversity and joining regions that enable diversification in identifying different host pathogens. In the generation of receptor diversity, there is a recombination of a V, D, and J segment of a beta or heavy chain, and a V and J segment for the alpha or light chain. For the BCR, this process helps expose very potent neutralizing antibodies that may be public in protecting against clinical disease. The generation of the variable regions may help guarantee higher levels of somatic mutation at the antigen binding site which may be shared or unique to an individual(s).

Despite the documented importance of lymphocyte receptors for antigen recognition and, hence, for the initiation of adaptive immune responses, the specific TCRs/BCRs that determine immunity to particular pathogens remain poorly understood. To our knowledge, no previous study has comprehensively mapped these receptors and analyzed how their expression profiles may correlate with individual variations in immune protection against malaria. In addition, the application of machine learning algorithms such as random forests, support vector machines may allow the identification of patterns on immune correlates that may predict protection against disease (118, 135).

Moreover, these approaches generate huge amounts of data that can be computationally analyzed to generate new, experimentally testable hypotheses. These may yield novel insights into the mechanisms underlying vaccine safety and efficacy. Importantly, data from such studies will inform pathways to which vaccine strategies should focus.

#### The B cell response and vaccine design

Effective vaccines are supposed to elicit and provide long-term protection as well as require both B and T cells to produce effective antibodies to neutralize surface-expressed antigens. B cell lineage vaccine design is an immune-focused approach that combines human immunology, structural biology, and computational protein design to develop a vaccine. The aim is to identify in both naïve and memory B cell receptors, paratopes (antigen binding sites) that interact with immunogens of interest. For a vaccine to be designed through this approach, memory B cell clones from the same lineage (or clone) are first identified and isolated from patients that produce broadly neutralizing antibodies or protective antibodies. These clones are then sequenced to obtain the V(D)J and VJ gene pairs that make up the B cell receptors in order to identify the paratope. Computational approaches are used to design an immunogen that interacts with the identified paratope (95). For *P. falciparum*, neutralizing antibodies produced by activated B cells are required to prevent the infection of new RBCs. By isolating such protective B cells from malaria patients and sequencing and analyzing their antigen receptors, it might be possible to identify immunogens able to induce protective immunity in susceptible individuals.

In malaria, the identification of broadly neutralizing antibodies remained elusive partly due to the high polymorphic nature of *P. falciparum* antigens. In addition, malaria vaccine candidates tend to induce antibodies with weak neutralizing ability, low breadth and strain-specific. However, Tan et al. (136) have recently identified monoclonal antibodies that can recognize *P. falciparum*-infected RBCs (iRBCs) from different strains of parasites. These antibodies recognize and bind to iRBCs through the RIFIN proteins, a group of variant antigens that are extracellularly expressed on the surface of iRBCs and have been associated with immune evasion (128) to initialize opsonization.

Another remarkable example is the identification of the novel antigenic target NPDP (part of the sequence in the N-terminal junction peptide) that is found between genes for the CSP and NANP and NVDP tandem repeats (137, 138). Independent studies in 2018 by Tan et al. (137) and Kisalu et al. (138) identified and isolated neutralizing antibodies from memory B cells and plasmablasts that could inhibit hepatocyte infection by PfSPZ. Through structural information, they were able to identify that these antibodies bind to conserved epitopes in the N-terminus of the CSP that is not found within the RTS,S vaccine.

Furthermore, mAbs that can inhibit parasite replication to about 97% have been isolated from CHMI donors immunized with RTS,S. Deciphering the structure and functionality of these antibodies have provided an informed overview on the structure of the CSP *in vivo*. Thus provides positive implications in the design of CSP immunogens against *P. falciparum* (139). However, there are still unsolved questions on the antibody responses to the PfCSP which have been described to be protective (139) and non-protective (129) as well as more structural information is needed to induce such potent neutralizing antibodies during vaccination Nonetheless, it is very interesting since they have implications in designing immunogens that can target specific immune responses and probably improve the efficacy of the RTS,S vaccine.

#### T cell response and vaccine design

Protective immunity to malaria liver-stage infection has been attributed to T cells in both human and rodent models. In studying immune responses to malaria such as cerebral malaria, murine models have provided significant understanding of various immunological properties that have impacted our understanding of the immune activity in humans.

For instance, Lau et al. (140) characterize MHC-restricted TCR that have potential in enhancing antigen presentation to T cells to enhance T cell immunity. They developed a novel CD8+ T cell receptor to *P. berghei* termed PbT-I from transgenic mouse with immune specificity for liver-stage and blood-stage infections. Isolated TCR genes from Vα8.3 and Vβ10 were isolated from a restricted hybridoma T cell line generated from *Plasmodium berghei* ANKA (PbA) blood-stage infection. Despite been developed for PbA, this transgenic MHC-I restricted T cell line was cross-reactive to *P. chabaudi* and *P. yoelli*. This implies that they may recognize conserved regions in rodent *Plasmodium spp*. Functional analysis revealed that the PbT-I cells produced effector cytokines (IFNγ, TNFα) and was positive for the degranulation marker (CD107a) showing their involvement in immune activity during the PbA infection. Using PbT-I CD8+ T cells, the peptides responsible for their activation were elucidated.

In a subsequent research, they identified and developed PbT-II CD4+ T cells from mouse transgenic line using the TCRα (Vα2.7, Jα12, Cα) and TCRβ gene (Vβ12, Dβ2, Jβ2.4) segments to blood-stage PbA infection (141). These cells were cross-reactive to rodent parasites (*P. berghei, yoelli and chabaudi*) and to *P. falciparum*. These MHC-II restricted PbT CD4+ T cells enhanced both humoral activity of B cells and cytotoxic activity of CD8+ T cells. In addition, the study confirmed that immunity to antigens in both blood stage and liver-stage development can restrict parasite replication in the hepatic stage and characterize the impact of blood stage antigen presentation to T cells that can enhance such T-cell immunity during infection. The uses of these target antigens may delineate protective immune responses and possibly circumvent pathologic outcomes. More importantly, further work should be focused on identifying and understanding such broadly reactive *Plasmodium*-specific T cells in host infections.

### Structure-based immunogen vaccine design

The structure-based vaccine approach can be employed in both the parasite and immune-focused approach. However, in the immune-focused approach, the principle is based on understanding the structural properties of the immune cell providing the desired response. Here, the properties of the antigenic binding site on the immune cell is studied at the atomic level (80, 81). By understanding these properties, the approach seeks to design and develop immunogens to target the protective response or develop these immune cells for use as interventions.

Structural-based vaccine design has aided in unmasking immunodominant epitopes in the haemagglutinin-stem of the influenza virus (142), the fusion protein in the respiratory syncytial virus (143) and CD4 binding site in HIV-1 virus. For example, identifying conserved immunogenic epitopes in HIV has been quite challenging. However, elucidating the structure of broadly neutralizing antibodies (bNAbs) has been very useful. Using NAb, subdominant epitopes in the CD4 binding site by the gp120 viral protein were identified. Probing the structure of the antigenic binding site on CD4, the structural properties helped in the development of a recombinant protein (RSC3) with specificity to the NAb. The RSC3 was further used to identify and isolate B cells that expressed broadly neutralizing antibodies with increased breadth. VRC01 and 3BNC117, highly potent monoclonal bnMAb with reactivity to about 91% to HIV-1 isolates were developed (144). Phase I clinical trials of the VRC01 were reported as safe with no allergenicity (145, 146). It is currently being evaluated in a Phase IIB trials with a projected overall efficacy of 53 and 82% (147). These observations indicate that using structural properties, subdominant epitopes can be uncovered to design immunogens to target a specific immune response.

Currently, there are few examples of the successful use of these approaches in malaria vaccine design. For instance, using invasion-inhibitory monoclonal antibodies, the novel structure of PfRh5 in complex with basigin was characterized, together with novel protective epitopes found in the complex (148). Similarly, for *P. vivax* infections, bNAbs that confer strain-specific immune responses (149) were isolated. These bNAbs, enabled the characterization of protective epitopes in the duffy binding protein that can be included in the design of a potent *P. vivax* vaccine (150).

## Conclusion

The development of a highly efficacious malaria vaccine faces many challenges, both technical and biological. Partly because the parasite is equipped with a variety of evasion mechanisms allow it to co-exist with the host. With the recent advent of high throughput approaches such as lymphocyte repertoire sequencing and structural design of immunogens, the breadth of protection of previous and current vaccine candidates may be enhanced as well as the identification of new candidate vaccines. In addition, vaccinologist may be able to design vaccines that drive the immune system through unusual yet protective pathways. Likewise, the application of mathematical modeling and computational approaches to the data thus obtained will open new pathways toward designing highly effective vaccines against malaria and aid in achieving the targets set by the malaria vaccine technology roadmap for 2030.

## Author contributions

All authors listed have made a substantial, direct and intellectual contribution to the work, and approved it for publication.

### Conflict of interest statement

The authors declare that the research was conducted in the absence of any commercial or financial relationships that could be construed as a potential conflict of interest.

## Abbreviations

ACTs: artemisinin-based combination therapies
PfEMP1: *P. falciparum* Erythrocyte Membrane Protein-1
MSP1: Merozoite Surface Protein
AMA1: Apical Membrane Antigen 1
Rh: Reticulocyte homolog (Rh)
MHC: Major histocompatibility complex
HLA: Human leukocyte antigen
PfSPZ: *Pf* sporozoite
PfCVac: *Pf* chemoprophylaxis vaccination
PfGAP: *Pf* genetically attenuated parasite
TBV: transmission-blocking vaccines
BCR: B cell receptor
bnMAb: broadly neutralizing monoclonal antibody
TCR: T cell receptor
VIMT: Vaccine that interrupt malaria transmission
SSM-VIMT, Sexual: sporogenic or mosquito stage VIMT
PE-VIMT: pre-erythrocytic VIMT.
